# Transcriptome-based identification and validation of reference genes for plant-bacteria interaction studies using *Nicotiana benthamiana*

**DOI:** 10.1038/s41598-018-38247-2

**Published:** 2019-02-07

**Authors:** Marina A. Pombo, Romina N. Ramos, Yi Zheng, Zhangjun Fei, Gregory B. Martin, Hernan G. Rosli

**Affiliations:** 1 Instituto de Fisiología Vegetal, INFIVE, Universidad Nacional de La Plata, CONICET, La Plata, Buenos Aires, Argentina; 2 Boyce Thompson Institute for Plant Research, 533 Tower Road, Ithaca, NY 14853 USA; 3 USDA-ARS Robert W. Holley Center for Agriculture and Health, Ithaca, NY 14853 USA; 4 Section of Plant Pathology and Plant-Microbe Biology, School of Integrative Plant Science, Cornell University, Ithaca, NY 14853 USA

## Abstract

RT-qPCR is a widely used technique for the analysis of gene expression. Accurate estimation of transcript abundance relies strongly on a normalization that requires the use of reference genes that are stably expressed in the conditions analyzed. Initially, they were adopted from those used in Northern blot experiments, but an increasing number of publications highlight the need to find and validate alternative reference genes for the particular system under study. The development of high-throughput sequencing techniques has facilitated the identification of such stably expressed genes. *Nicotiana benthamiana* has been extensively used as a model in the plant research field. In spite of this, there is scarce information regarding suitable RT-qPCR reference genes for this species. Employing RNA-seq data previously generated from tomato plants, combined with newly generated data from *N. benthamiana* leaves infiltrated with *Pseudomonas fluorescens*, we identified and tested a set of 9 candidate reference genes. Using three different algorithms, we found that *NbUbe35*, *NbNQO* and *NbErpA* exhibit less variable gene expression in our pathosystem than previously used genes. Furthermore, the combined use of the first two is sufficient for robust gene expression analysis. We encourage employing these novel reference genes in future RT-qPCR experiments involving *N. benthamiana* and *Pseudomonas* spp.

## Introduction

Plants are in constant interaction with beneficial and pathogenic microorganisms. For detection of these microbes, plants use pattern-recognition receptors (PRRs) that perceive conserved features named microbe- (or pathogen-) associated molecular patterns (MAMPs or PAMPs), activating pattern-triggered immunity (PTI), the first layer of inducible plant defense^1–3^. PTI is associated with the production of reactive oxygen species, activation of mitogen-activated protein kinases (MAPKs), changes in intracellular calcium concentrations and changes in gene expression that prevent the infection of many potentially pathogenic microbes^4–8^. Some bacterial pathogens such as *Pseudomonas syringae* use a type III secretion system to introduce virulence proteins (effectors) into the plant cell cytoplasm to counteract PTI^5,9^ and to manipulate host metabolic processes in order to facilitate growth and proliferation in the apoplast^10,11^. The second layer of plant immune response, referred as effector-triggered immunity (ETI), consists of the intracellular detection of pathogen effectors by resistance proteins (R proteins)^12,13^. This immune response is often associated with a hypersensitive response (HR) that leads to localized cell death, which restricts pathogen spread^5,14^. Some effectors are involved in the suppression of this plant-immunity associated cell death^15^.

Pathogens cause up to 30% of crop loss, which has a detrimental economical impact^16^. Therefore, scientific progress aimed at understanding how plants respond to infections is an important step for the design of new technologies to increase food production and quality. *Pseudomonas syringae* pv. *tomato* (*Pst*) has been used as a model bacteria in the molecular studies of plant-pathogen interactions^17^. One of the reasons for this is that *Pst* can be manipulated to infect tomato, *Arabidopsis* and also *Nicotiana benthamiana* plants^17^.

The Australian endemic plant *N. benthamiana*, is an important model organism in plant biology^18^. This species belongs to the Solanaceae family along with several economically important crops such as tomato, eggplant, potato, tobacco and petunia. It was adopted for virology studies because of its susceptibility to different virus strains^19^. Nowadays, several reasons make this species a model for plant research, including amenability to genetic transformation, high efficiency using virus-induced gene silencing (VIGS) and efficient transient protein expression and the availability of a draft genome sequence^18–20^.

Several experimental methods are available for gene expression quantification. Reverse transcription-quantitative PCR (RT-qPCR) is a widely used technique for the determination of mRNA level changes in different biological systems^21^. Although it is considered an important and frequently used method in laboratories, RT-qPCR results can be misinterpreted if several critical steps are not carefully followed^22,23^. One of them is the selection of optimal reference genes for accurate normalization of transcript abundance. A reference gene should have a minimal expression variation in the analyzed conditions^23,24^. Most of the RT-qPCR reference genes traditionally used such as glyceraldehyde-3-phosphate dehydrogenase (*GADPH*), 18 S ribosomal RNA (*18 S RNA*), beta-tubulin-4 (*TUB4*), elongation factor 1 alpha (*EF1α*), polyubiquitin (*UBQ*), actin (*ACT*), have been adopted from Northern blot and semi-quantitative RT-PCR experiments^24^. However, recent studies indicate that traditional reference genes are not always stably expressed and suggest the necessity of a systematic selection and validation of reference genes for each particular experimental condition. Despite the importance of *N. benthamiana* as a research model plant, to our knowledge only two studies have evaluated reference genes for this species. These reports analyzed the expression stability of traditional reference genes in plants infected with virus^25^ or plants used for VIGS experiments^26^.

RNA-seq is a powerful high-throughput technology used for transcriptome analysis in different organisms under diverse conditions and treatments^27–30^. Previously, RNA-seq was used to study transcriptional changes during the activation of PTI in tomato and the subsequent inhibition of this response by *Pst* AvrPto and AvrPtoB effectors^7^. Following a similar approach, genes specifically induced or repressed during PTI or ETI activation were identified^8^. Analyzing this large set of data, combined with newly generated data, novel RT-qPCR reference genes in the tomato-*Pseudomonas* pathosystem were recently identified and validated^31^. Taking advantage of these data previously generated for tomato, we aimed here at transferring the information to *N. benthamiana* by identifying the most closely related genes, with the hypothesis that these genes would also have stable expression in *N. benthamiana*. We generated new RNA-seq data for *N. benthamiana* challenged with *Pseudomonas fluorescens* 55 (PTI activation) and used this information to establish a set of candidate genes for validation. These novel reference genes were then tested using three different algorithms (geNorm, NormFinder and Bestkeeper) and their performance compared with two traditional reference genes (*NbEF1α* and *NbGADPH*) and *NbPP2a*, a previously validated reference gene for virus-infected *N. benthamiana*^25^. Our analysis allowed the identification of three novel RT-qPCR reference genes (*NbUbe35*, *NbNQO* and *NbErpA*) that can be used in the *N. benthamiana*-*Pseudomonas* pathosystem or related systems.

## Results

### Selection of stably expressed genes based on tomato and *Nicotiana benthamiana* RNA-seq data

In order to identify genes with low expression variation to be used as reference in RT-qPCR experiments, we took advantage of a previous analysis of gene stability based on RNA-seq data from tomato leaves with different treatments (37 treatments/time points with an average of 3 biological replicates generated in independent experiments)^31^. This large set of tomato data was narrowed down to 50 genes with the most stable expression across all treatments. Performing BlastX analysis using these tomato genes as input and *N. benthamiana* proteins as database, we identified the closest putative orthologs and hypothesized they would also be stably expressed genes. To assist in the selection of reference genes, we performed RNA-seq analysis with *N. benthamiana* leaves vacuum-infiltrated with a suspension of *Pseudomonas fluorescens* 55 and MgCl_2_ as a mock. This bacterial treatment, that results in a strong PTI induction and large transcriptomic changes at 6 h after infiltration (hai)^7^, allowed the identification of 10,300 differentially expressed genes from the 57,139 predicted genes in *N. benthamiana* (Supplementary Table S1). Using this information generated from *N. benthamiana* tissue, we calculated the coefficient of variation (CV) of the 50 selected genes, using the expression values (RPKMs, reads per kilobase of transcript per million mapped reads) of each biological replicate individually. The lower the CV is, the more stable the expression of the gene is across the conditions. In this way we were able to select 9 genes with the lowest CV (ranging from 5.14% to 10.27%) for analysis (Supplementary Table S2). We also selected a gene named *NbPP2a* (Niben101Scf09716g01002.1) previously validated as a stable reference gene in virus-infected *N. benthamiana* plants^25^ and two traditional plant reference genes *NbEF1α* and *NbGADPH*.

### Analysis of candidate reference genes expression profiles indicated high efficiencies and unique transcript amplifications

We evaluated the amplification efficiencies of each selected gene performing RT-qPCR using cDNA dilutions (1:5, 1:10, 1:100, 1:1000). Amplification efficiency E was measured as 10^−1/slope^ and expressed in percentage (Supplementary Table S3). All the primers showed high E values ranging from 92% to 100%. We also analyzed the specificity of the amplification through melting curves for all pairs of primers used and in all cases observed a single peak accounting for a single PCR product (Supplementary Fig. S1). We checked the presence of contamination and primer dimers with the analysis of the non-template control melting curves. Only in one of the three technical replicates corresponding to *NbTspan* gene, we observed a small peak. Analysis in detail of its corresponding Cq value (35.25) showed that was nearly 10 Cq-values lower than those obtained when using template (<25.80). Considering the mentioned difference in Cq values, this amplification was ignored^22^.

### Cycle amplification values (Cq) allowed narrowing down the number of selected genes

We designed an experiment for the evaluation of our set of genes under different immune responses using the model plant *N. benthamiana*. A summary of the infiltrations performed is shown in Table 1. In order to induce PTI activation we infiltrated *N. benthamiana* leaves with *Pseudomonas fluorescens* 55 (*Pf*)^32^ and used 10 mM MgCl_2_ as a mock treatment. Additionally, we infiltrated *N. benthamiana* leaves with *Pst* DC3000^33^ and *Pst* DC3000 Δ*hopQ1-1*^34^. The comparison of these last two treatments allows dissecting ETI response. We collected leaf tissue from 3 biological replicates at 6 and 12 hai and later on we visually monitored the development of symptoms on the plants to confirm activation of the expected defense responses.

**Table 1:** Summary of the experiments performed.

Plant	Inoculum	Concentration	Immune response evaluated	Time points	Experiment
*Nicotiana benthamiana*	MgCl_2_	10 mM	PTI	6 h	RNA-seq
	*Pseudomonas fluorescens* 55	10^8^ cfu/ml			
	MgCl_2_	10 mM	PTI	6, 12 h	Validation of RT-qPCR reference genes
	*Pseudomonas fluorescens* 55	10^8^ cfu/ml			
	*Pst* DC3000^a^	5 × 10^6^ cfu/ml	ETI		
	*Pst* DC3000 Δ*hopQ1-1*^b^				

^a^*Pseudomonas syringae pv. tomato* (*Pst*) DC3000.

^b^*Pseudomonas syringae* pv. *tomato* (*Pst*) DC3000 mutant, lacking HopQ1-1 effector.

**Figure 1:**
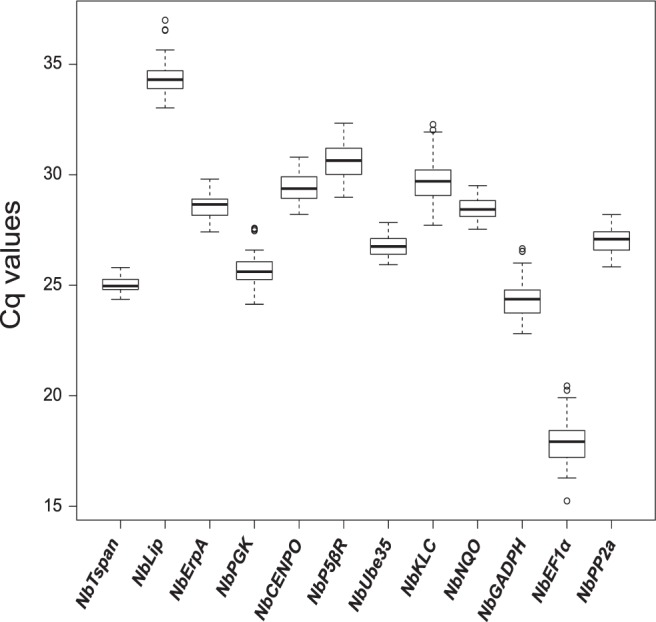
Cycle quantification (Cq) values of selected genes. Box and whisker plot graph showing Cq values of each selected gene in all treatments (Table 1) for all the samples analyzed (three biological replicates per treatment, three technical replicates per sample, n = 24). Black lines and boxes represent the Cq medians and 25/75 percentiles, respectively. Whisker caps represent the minimum and maximum Cq values. ○, indicate outliers.

Average Cq values (Fig. 1) for most of the genes were within the recommended values for a RT-qPCR reference gene (higher than 15 and lower than 30)^35^. Two genes were the exception, with mean Cq values of 34.4 (*NbLip*) and 30.6 (*NbP5βR*), and were consequently excluded from further analysis. Among the 10 remaining genes, *NbEF1α* and *NbTspan* had the largest (5.2) and smallest (1.4) difference between maximum and minimum Cq values, respectively.

### Based on different algorithms, three newly identified reference genes are the most stably expressed

**Figure 2:**
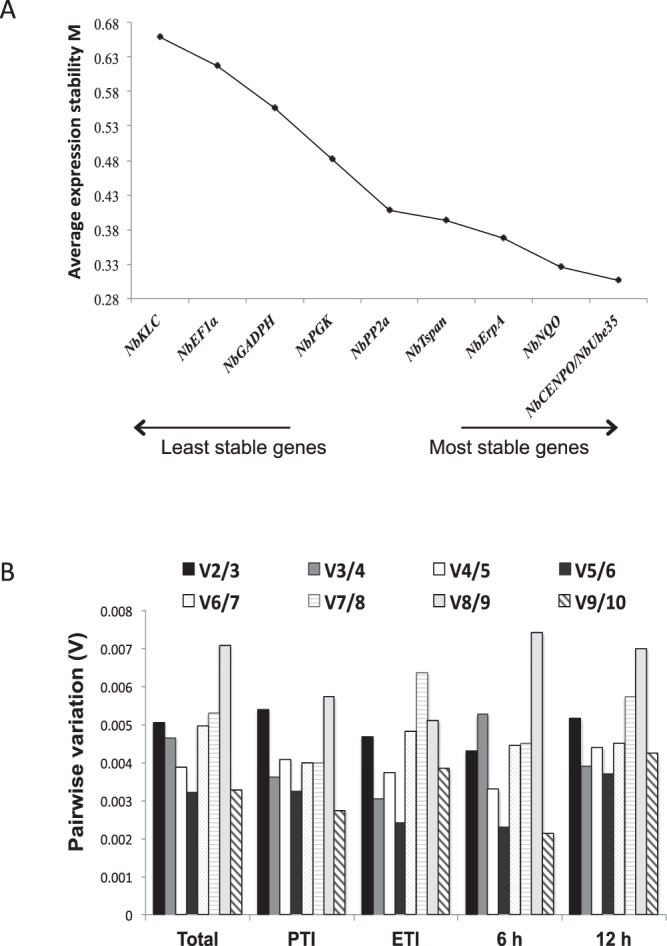
geNorm analysis of selected reference genes in *N. benthamiana* leaves infiltrated with different *Pseudomonas* strains. (**A**) *N. benthamiana* reference genes were ranked based on expression stability calculated by geNorm. M values represent the average pairwise variation of the gene compared with all other control genes. (**B**) Pairwise variation (Vn/Vn + 1) for determination of the optimal number of reference genes. The pairwise variation was calculated considering all the samples treatments and time-points together (Total), mock and *P. fluorescens* (PTI), *Pst* DC3000 and *Pst* DC3000 Δ*hopQ1-1* (ETI), samples taken at 6 hai (6 h) or samples taken at 12 hai (12 h).

To estimate gene expression stability, we analyzed RT-qPCR data with three different software tools. We first used geNorm software^36^ to establish the average expression stability value M. This program determines the pairwise variation of each gene with all other analyzed genes under the same experimental conditions. The lower the M value, the more stable the gene is. Three genes presented the highest variability, with M values over the usually proposed cutoff value of M ≤0.5^37^. These genes were *NbKLC* (M = 0.659), *NbEF1α* (M = 0.618) and *NbGADPH* (M = 0.556). This software also selects an optimal pair of reference genes and in this experiment the most stable ones were *NbCENPO* and *NbUbe35* with M value of 0.306 (Fig. 2A).

We then estimated the minimal number of reference genes to be used. To achieve this, we determined the pairwise variation (V) of a normalization factor (NF) calculated by introducing reference genes one by one, starting from the two least variable and adding the rest in a decreasing stability order until the whole set was included^36^. We decided to analyze our data as a whole, only including PTI activation (*Pf* 55 and mock), only including ETI activation (*Pst* DC3000 and *Pst* DC3000 Δ*hopQ1-1*), only including samples taken at 6 hai (6 h) and only including samples taken at 12 hai (12 h) (Fig. 2B). Regardless of using the complete dataset, the defense response or time-point subsets, the results were very similar. In all of the cases, the V2/3 value obtained was smaller than the proposed cut-off of 0.15^36^, suggesting that only the two most stable reference genes (*NbCENPO* and *NbUbe35*) identified by geNorm software are sufficient for a good normalization of RT-qPCR data, regardless of the type of response evaluated (PTI or ETI) or time-point (6 or 12 h).

**Figure 3:**
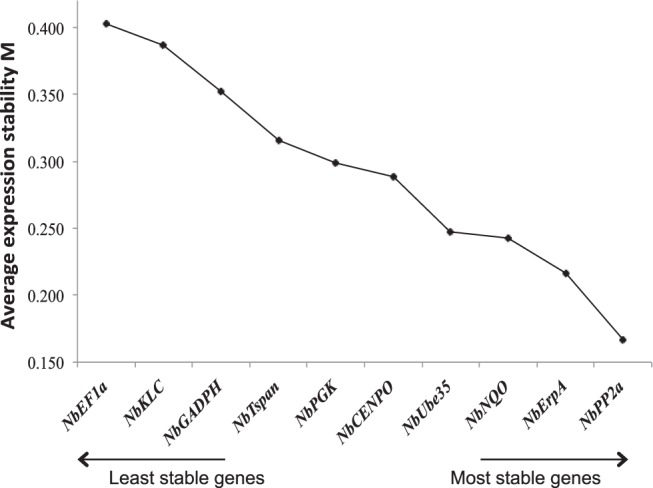
NormFinder expression stability of selected reference genes in *N. benthamiana*-*Pseudomonas* pathosystem. *N. benthamiana* reference genes were ranked based on expression stability calculated by NormFinder. The analysis was performed using expression data from all biological replicates and treatments (n = 24).

Another algorithm that also calculates an M index is NormFinder^38^. To calculate this index, NormFinder estimates the intragroup (within each sample/treatment) and then the intergroup (within different groups of samples/treatments) variation. Similarly to geNorm analysis, NormFinder selected *NbEF1α*, *NbKLC* and *NbGADPH* as the most variable genes, with M values of 0.402, 0.387 and 0.352, respectively. However, the most suitable reference genes derived from NormFinder analysis were *NbPP2a* (M = 0.167), *NbErpA* (M = 0.216) and *NbNQO* (M = 0.242) (Fig. 3).

To further analyze the candidate gene stability, we used BestKeeper^39^. This tool allows the analysis of 10 genes in two steps. First, it calculates different statistical parameters and then, a coefficient of correlation (*r*) is obtained by comparing a BestKeeper index with each particular gene (Table 2). According to standard deviation (SD) values all the genes under study were suitable to be considered as reference genes (SD [±Cq] <1 and SD [±x-fold] <2)^39^. We analyzed their variation parameters (SD [±Cq] and CV [% Cq]), and observed that *NbTspan*, *NbUbe35* and *NbNQO* were the most stable genes and *NbEF1α*, *NbGADPH* and *NbKLC*, the most variable ones. The calculated *r* values for the comparison of each gene with the BestKeeper index were inconsistent with the SD-based analysis described above. For example, according with SD [±Cq] value, *NbEF1α* is the most variable gene but its coefficient of correlation was high (0.81). We therefore used SD [±Cq] and CV [% Cq] parameters to rank the genes based on their stability (Table 2). This approach is employed in the RefFinder tool^40^ as a ranking method for the BestKeeper output.

**Table 2:** Analysis of ten selected *N. benthamiana* reference genes using Bestkeeper algorithm.

Ranking	1	2	3	4	5	6	7	8	9	10
Gene name	*NbTspan*	*NbUbe35*	*NbNQO*	*NbErpA*	*NbPP2a*	*NbCENPO*	*NbPGK*	*NbKLC*	*NbGADPH*	*NbEF1α*
Geo Mean [Cq]	25.02	26.75	28.46	28.58	27.02	29.45	25.57	29.69	24.31	17.86
Min [Cq]	24.37	25.93	27.54	27.41	25.83	28.21	24.14	27.72	22.81	15.24
Max [Cq]	25.80	27.83	29.51	29.80	28.20	30.80	27.58	32.27	26.65	20.44
**SD [ ± Cq]**	**0.28**	**0.39**	**0.41**	**0.42**	**0.45**	**0.53**	**0.53**	**0.69**	**0.70**	**0.74**
**CV [% Cq]**	**1.13**	**1.44**	**1.46**	**1.47**	**1.67**	**1.81**	**2.05**	**2.34**	**2.87**	**4.12**
Min [x-fold]	−1.57	−1.71	−1.83	−2.14	−2.25	−2.33	−2.69	−3.65	−2.79	−5.61
Max [x-fold]	1.71	2.03	1.98	2.23	2.24	2.49	4.03	5.48	5.03	5.45
**SD [ ± x-fold]**	**1.21**	**1.30**	**1.33**	**1.34**	**1.37**	**1.44**	**1.44**	**1.61**	**1.62**	**1.66**
**Coeff. of corr. [r]**	0.33	0.70	0.69	0.78	0.88	0.58	0.84	0.76	0.78	0.81
*p*-value	0.004	0.001	0.001	0.001	0.001	0.001	0.001	0.001	0.001	0.001

[Cq], quantification cycle; Geo Mean [Cq], geometric mean of Cq; Min and Max [Cq], the extreme values of Cq; SD [Cq], standard deviation of Cq; CV [%Cq], coefficient of variance expressed as a percentage on the Cq level; Min [x-fold] and Max [x-fold], the extreme values of expression levels expressed as an absolute x-fold over or under regulation coefficient; SD [±x-fold], standard deviation of the absolute regulation coefficients, Coeff. of corr [r], coefficient of correlation between each candidate and the BestKeeper index.

When we compared the outputs of the statistical programs used, we observed a certain degree of discrepancy mainly in the selection of the genes with lower variability across the experiment. This degree of divergence among the stability ranking generated by geNorm, NormFinder and BestKeeper has been previously reported and could attributed to the fact that these tools are based on different algorithms^31,41–43^. In order to analyze the results globally, we calculated the arithmetical mean of the ranking value obtained for each gene using all three algorithms^31,41,42,44^. As a result, *NbUbe35* was rated as the most stable with a mean ranking value of 2.33 (Table 3). With this overall stability ranking we reanalyzed the pairwise variation in order to establish the minimum number of reference genes for normalization, considering the proposed threshold of 0.15^36^. Based on this analysis, the combination of *NbUbe35* and *NbNQO* is sufficient for accurate normalization (Supplementary Fig. S2) and we consequently used this combination for further experiments.

**Table 3:** Gene stability ranking established by the combination of geNorm, NormFinder and BestKeeper results.

Global ranking	Gene	geNorm	NormFinder	BestKeeper	Mean
1	*NbUbe35*	1	4	2	2.33
2	*NbNQO*	3	3	3	3.00
3	*NbErpA*	4	2	4	3.33
4	*NbPP2a*	6	1	5	4.00
5	*NbCENPO*	1	5	6	4.00
6	*NbTspan*	5	7	1	4.33
7	*NbPGK*	7	6	7	6.67
8	*NbGADPH*	8	8	9	8.33
9	*NbKLC*	10	9	8	9.00
10	*NbEF1α*	9	10	10	9.67

### Validation of the selected genes confirmed their suitability as reference genes

**Figure 4:**
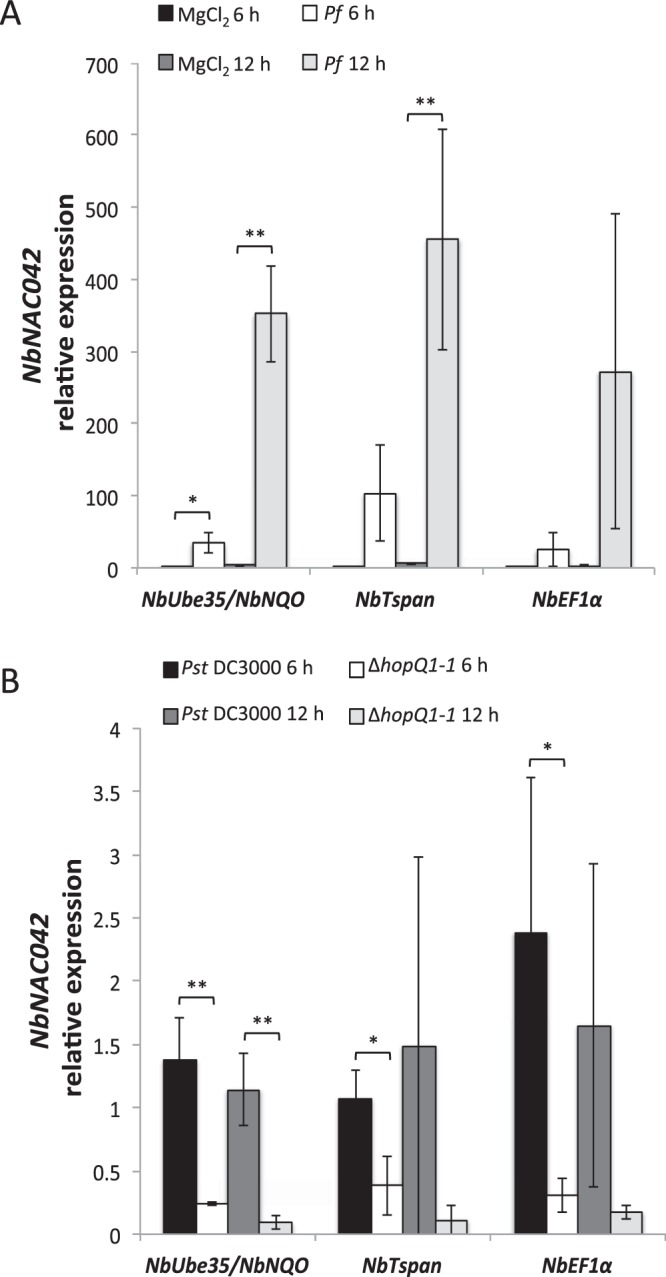
Relative expression of *NbNAC042* analyzed using different reference genes. Relative expression analysis by RT-qPCR at two time points (6 and 12 hai) using plants infiltrated with: (**A**), mock (10 mM MgCl_2_) or 10^8^ cfu/ml *Pseudomonas fluorescens* 55 (*Pf*); (**B**), 5 × 10^6^ cfu/ml of *Pseudomonas syringae* pv. *tomato* DC3000 (*Pst* DC3000) or *Pst* DC3000 Δ*hopQ1-1* (Δ*hopQ1-1*) strains. In both cases, the geometric mean of the two best (*NbUbe35/NbNQO*), the intermediate (*NbTspan*) or the worst (*NbEF1α*) reference genes was used for normalization of the data. The relative expression was expressed as E^−ΔΔCq^, where E corresponds to the primer efficiency value. Calibration samples are 10 mM MgCl_2_ 6 h in A and *Pst* DC3000 6 h in B (biological replicate 1 in both cases). Bars represent the mean of three biological replicates and three technical replicates with their corresponding standard deviation. ** or * indicate significant differences using Student *t-test* with *p*-values < 0.01 or <0.05, respectively.

Using BlastP we identified two putative *N. benthamiana* orthologs of a previously described tomato gene (Solyc02g069960) that is induced by PTI^8^: Niben101Scf04323g01009.1 and Niben101Ctg15860g00004.1 (*NbNAC042*). According to our RNA-seq data the first one had very low expression (0-0.46 RPKM), while *NbNAC042* was induced by PTI with RKPM values ranging between 0 and 20.53 (Supplementary Table S4). For this reason we chose *NbNAC042* to put to test the two most stable genes described here (*NbUbe35* and *NbNQO*) and compare their performance to a middle-ranked gene (*NbTspan*) and a traditionally used reference gene (*NbEF1α*). Regardless of the reference genes used the trend of transcript abundance increase of *NbNAC042* upon PTI induction (6 and 12 hai), was similar (Fig. 4A). Nevertheless, the combined use of *NbUbe35* and *NbNQO* resulted in lower standard deviation values allowing establishing statistically significant differences at both time-points and at a lower significance level. To our surprise *NbNAC042* gene expression increased with ETI activation (*Pst* DC3000 vs. *Pst* DC3000 Δ*hopQ1-1*), given Solyc02g069960 in tomato is not affected by ETI at 6 hai^8^. Again, the use of the combination of *NbUbe35* and *NbNQO* lead to lower deviations and lower significance levels (Fig. 4B). These results highlight the relevance of the selection of accurate reference genes for the experimental system under study.

## Discussion

Due to its amenability to genetic transformation, virus induced gene silencing and transient protein expression, *Nicotiana benthamiana* has become very popular in the plant biology field^18^. Particularly, its susceptibility to a wide variety of pathogens made this model plant one of the most widely used in molecular studies of plant-pathogen interactions^19^. RT-qPCR is a frequently used technology for detection and quantification of gene expression, but accurate data interpretation highly depends on the use of appropriate reference genes whose expression should have minimal variations in the tissue, treatment or condition to be analyzed^35^. In this sense, several reports have contributed to the development of reference genes for the analysis of plant gene expression in the interaction with different pathogens. Some examples are tomato-virus^41^, tomato-bacteria^31^, wheat-fungus^45^, soybean-nematode and insect^46^ and rice-virus^47^ interactions. In spite of the extensive use of *N. benthamiana*, to our knowledge there are only two reports that analyzed the expression stability of traditional reference genes for plants infected with virus^25^ and for VIGS experiments^26^.

RNA-seq has become a powerful technology used for transcriptomic analysis in different organisms and treatments^27,28,48^. The information generated using this technique was used in the plant research field for the selection of new and more robust RT-qPCR reference genes in grape, soybean, potato, *Lycoris* and tomato^31,49–52^. In this work we used a new approach for the selection of novel stably expressed genes that can be used in *N. benthamiana*-bacterial interaction studies. We have taken advantage of previously generated tomato RNA-seq information^7,8,31^. These studies include transcriptional changes of tomato leaves for studying PTI and ETI activation and the influence of bacterial effectors on plant defenses, through the infiltration of MAMPs and bacterial strains and mutants along with untreated tomato plants. Together these experiments constitute a robust and large set of data that allowed the identification of novel reference genes in the tomato-*Pseudomonas* pathosystem^31^. Due to the closeness between the two species, we were able to find the *N. benthamiana* gene orthologs of the stably expressed tomato genes previously reported. This strategy was earlier useful to find reference genes in pepper using microarray information generated from tomato^53^. In this work we produced new RNA-seq data from *N. benthamiana* leaves infiltrated with *Pseudomonas fluorescens* 55, whose comparison with a mock treatment, accounts for a strong transcriptional PTI induction^7^. In agreement with this, we were able to identify 10,300 genes differentially expressed in *N. benthamiana* when challenged with this strain (Supplementary Table S1), This new dataset assisted in the selection of 9 novel genes with low coefficient of variation in the *N. benthamiana*-*Pseudomonas* pathosystem. This strategy led to the identification of a set of stably expressed genes in *N. benthamiana* that mostly differs from those previously found in tomato by an RNA-seq approach^31^, further confirming that it is necessary to evaluate reference genes for every system.

Using different algorithms such as geNorm^36^, NormFinder^38^ and BestKeeper^39^ we identified 3 reference genes (*NbUBE35*, *NbNQO*, *NbErpA*) that are more stably expressed compared to the commonly used ones that we selected for comparison (*NbEF1α* and *NbGAPDH*). We strongly recommend the use of *NbUbe35* jointly with *NbNQO* as reference genes for expression studies that involve *N. benthamiana* leaf tissue infiltrated with *Pseudomonas* spp. or related experiments. According to our pairwise variation analysis, the use of two of these genes is sufficient to obtain accurate results (Supplementary Fig. S2). We also included in our analysis the *NbPP2a* gene, previously found to be the most stably expressed gene in *N. benthamiana*-virus interactions^25^. Although this gene performed fairly well in our study system, we found 3 genes whose overall expression was more stable (Table 3). When we put to test the combination of *NbUbe35*/*NbNQO* against a middle-ranked gene (*NbTspan*) or *NbEF1α* as reference, we found discordant results in terms of the capability of identifying statistically significant differences. Although the expression trend was similar, the high standard deviation obtained using either *NbTspan* or *NbEF1α* as reference prevented the detection of significant differences between the samples (Fig. 4). These results highlight the importance of the selection and validation of reliable reference genes for an accurate interpretation of the results. We therefore encourage the use of the information generated in this work in future RT-qPCR experiments involving *N. benthamiana*-*Pseudomonas* spp. pathosystems.

## Material and Methods

### Bacterial strains and growth conditions

Bacterial strains used were: *Pseudomonas fluorescens* 55 (*P. fluorescens*)^32^, *Pseudomonas syringae* pv. *tomato* (*Pst*) DC3000^33^, *Pst* DC3000 Δ*hopQ1-1*^34^. All of them were grown on King’s B medium at 30 °C. Antibiotics used were: ampicillin (100 μg/ml) for *P. fluorescens* and rifampicin (10 μg/ml) for *Pst* DC3000 and mutants.

### Plant material and treatments

RNA-seq analysis was performed using 6-week old *Nicotiana benthamiana* plants vacuum-infiltrated with 10^8^ cfu/ml *P. fluorescens* suspension or 10 mM MgCl_2_ as a mock treatment. Leaves were sampled at 6 h after infiltration (hai), frozen in liquid N_2_ and stored at −80 °C until processed.

For RT-qPCR studies, 6-week old *N. benthamiana* leaves were syringe-infiltrated with a suspension of 10^8^ cfu/ml *P. fluorescens*, 5 × 10^6^ cfu/ml *Pst* DC3000, 5 × 10^6^ cfu/ml *Pst* DC3000 Δ*hopQ1-1* or 10 mM MgCl_2_. Leaf samples were collected at 6 and 12 hai, frozen in liquid N_2_ and stored at −80 °C until processed.

In all the experiments, three biological replicates per infiltration were used. Details of the experiments are shown in Table 1.

### RNA-seq library preparation and analysis

Total RNA was isolated using TRIzol reagent (Life Technologies, NY, USA) and libraries prepared as described previously^8^. Barcoded libraries were multiplexed by 12 in each lane and sequenced on an Illumina HiSeq 2000 equipment with 101 bp pair-end read mode. Sequence reads generated in this work have been deposited in the NCBI sequence read archive (SRA) under accession number SRP118889. Analysis of the RNA-seq data was performed as described previously^8^.

### Selection of the candidate genes and primer design

Taking advantage of tomato gene expression stability ranking previously generated based on RNA-seq data^31^, using BlastX analysis from Sol Genomics Network^54^, we identified 50 *N. benthamiana* orthologs which we hypothesized had stable gene expression. Using the newly generated RNA-seq data (*N. benthamiana* leaves challenged with *P. fluorescens* 55) we discarded those with low expression (RPKM <3) and ranked them based on their coefficient of variation (CV) across treatments and biological replicates. We then selected the 9 most stably expressed genes for validation (Supplementary Table S2). Additionally, two traditional reference genes used in *N. benthamiana* RT-qPCR experiments (*NbGADPH* and *NbEF1α*) and *NbPP2a*, the most stably expressed gene identified in a previous report using virus-infected *N. benthamiana* plants^25^, were included for the analysis.

We used BlastP to identify two putative *N. benthamiana* orthologs of a previously reported tomato gene (Solyc02g069960) that is induced by PTI^8^. From the two closest found, Niben101Scf04323g01009.1 and Niben101Ctg15860g00004.1 (*NbNAC042*), we selected *NbNAC042* based on its gene expression level and PTI induction (Supplementary Table S4), to test the performance of the most stable reference genes described in this work.

The nucleotide sequence of each gene was downloaded from the Sol Genomics Network webpage^54^ and primers were designed using PrimerQuest tool (Integrated DNA Technologies). Primer efficiencies were checked by RT-qPCR using different cDNA dilutions. This information, along with the accession number of all *N. benthamiana* genes used in this work, is show in Supplementary Table S3. Dissociation curves were performed to confirm amplification specificity (Supplementary Fig. S1).

### RNA isolation and cDNA preparation

Total RNA was isolated using the Tri-Reagent (Sigma Aldrich) following the manufacturer’s instructions. RNA integrity was assayed by 1% agarose gel electrophoresis. Total RNA (8 μg) was processed with RQ1 RNase-free DNase (Promega) for 60 minutes at 37 °C to eliminate potential DNA contamination and then purified using a chloroform:octanol mix (24:1). RNA concentration and purity was determined using a CLARIOstar microplate reader (BMG Labtech). Purified RNA (2.4 μg) was used to prepare cDNA using M-MLV reverse transcriptase (Promega) with random primers according to the manufacturer’s instructions.

### RT-qPCR reactions

RT-qPCR was performed as described previously^55^ in 96-well plates (Thermo Fisher Scientific) on a StepOnePlus system (Applied Biosystems). Primer sequences and characteristics are shown in Supplementary Table S3. The reaction mix was performed using: 5 μl of FastStart Universal SYBR Green Master (Rox) (Roche Life Sciences), 2 μl of 2 μM primer mix, 2 μl of a diluted 1:10 cDNA and water to complete a final volume of 10 μl. Cycling conditions were 95 °C for 10 min, and 40 cycles of 95 °C for 15 s, 60 °C for 1 min. All RT-qPCR experiments were performed using three biological and three technical replicates.

### Evaluation and validation of reference gene expression stability

Data obtained from the RT-qPCR experiments were analyzed using three statistical programs: geNorm^36^, NormFinder^38^ and BestKeeper^39^. The relative expression of *NbNAC042* gene was expressed as E^−ΔΔCq^, where E corresponds to the primer efficiency value. When a pair of reference genes were used (*NbUbe35*/*NbNQO*) the geometric Cq mean and efficiency average were employed.

## Supplementary information

Supplementary information

Supplementary Table S1

Supplementary Table S2

Supplementary Table S3

Supplementary Table S4

## References

[1] Boller T, He SY (2009). Innate immunity in plants: an arms race between pattern recognition receptors in plants and effectors in microbial pathogens. Science.

[2] Schwessinger B, Ronald PC (2012). Plant innate immunity: perception of conserved microbial signatures. Annu Rev Plant Biol.

[3] Couto D, Zipfel C (2016). Regulation of pattern recognition receptor signalling in plants. Nat Rev Immunol.

[4] Segonzac C, Zipfel C (2011). Activation of plant pattern-recognition receptors by bacteria. Curr Opin Microbiol.

[5] Dodds, P. N. & Rathjen, J. P. Plant immunity: towards an integrated view of plant-pathogen interactions. *Nat Rev Genet* **11** (2010). 10.1038/nrg2812 20585331

[6] Monaghan J, Zipfel C (2012). Plant pattern recognition receptor complexes at the plasma membrane. Curr Opin Plant Biol.

[7] Rosli H (2013). Transcriptomics-based screen for genes induced by flagellin and repressed by pathogen effectors identifies a cell wall-associated kinase involved in plant immunity. Genome Biol.

[8] Pombo MA (2014). Transcriptomic analysis reveals tomato genes whose expression is induced specifically during effector-triggered immunity and identifies the Epk1 protein kinase which is required for the host response to three bacterial effector proteins. Genome Biol.

[9] Feng F, Zhou JM (2012). Plant-bacterial pathogen interactions mediated by type III effectors. Curr Opin Plant Biol.

[10] Macho AP (2016). Subversion of plant cellular functions by bacterial type-III effectors: beyond suppression of immunity. New Phytol.

[11] Toruño, T. Y., Stergiopoulos, I. & Coaker, G. Plant pathogen effectors: cellular probes interfering with plant defenses in spatial and temporal manners. *Annu Rev Phytopathol* **54**, 10.1146/annurev-phyto-080615-100204 (2016). 10.1146/annurev-phyto-080615-100204 PMC5283857 27359369

[12] Moffett P (2009). Mechanisms of recognition in dominant R gene mediated resistance. Adv Virus Res.

[13] Maekawa T, Kufer TA, Schulze-Lefert P (2011). NLR functions in plant and animal immune systems: so far and yet so close. Nature Immunol.

[14] Tsuda K, Sato M, Stoddard T, Glazebrook J, Katagiri F (2009). Network properties of robust immunity in plants. PLoS Genet.

[15] Wei H-L, Zhang W, Collmer A (2018). Modular study of the type III effector repertoire in *Pseudomonas syringae* pv. *tomato* DC3000 reveals a matrix of effector interplay in pathogenesis. Cell Rep.

[16] Jones, J. D. G., Vance, R. E. & Dangl, J. L. Intracellular innate immune surveillance devices in plants and animals. *Science* **354**, 10.1126/science.aaf6395 (2016). 10.1126/science.aaf6395 27934708

[17] Xin X-F, He SY (2013). *Pseudomonas syringae* pv. *tomato* DC3000: a model pathogen for probing disease susceptibility and hormone signaling in plants. Annu Rev Phytopathol.

[18] Todesco M, de Felippes FF (2016). Why benthamiana went viral. Trends Plant Sci.

[19] Goodin MM, Zaitlin D, Naidu RA, Lommel SA (2008). *Nicotiana benthamiana*: its history and future as a model for plant-pathogen interactions. Mol Plant-Microbe Interact.

[20] Bombarely A (2012). A draft genome sequence of *Nicotiana benthamiana* to enhance molecular plant-microbe biology research. Mol Plant-Microbe Interact.

[21] Bustin S (2002). Quantification of mRNA using real-time reverse transcription PCR (RT-PCR): trends and problems. J Mol Endocrinol.

[22] Bustin SA (2009). The MIQE guidelines: minimum information for publication of quantitative real-time PCR experiments. Clin Chem.

[23] Derveaux S, Vandesompele J, Hellemans J (2010). How to do successful gene expression analysis using real-time PCR. Methods.

[24] Huggett J, Dheda K, Bustin S, Zumla A (2005). Real-time RT-PCR normalisation; strategies and considerations. Genes Immun.

[25] Liu D (2012). Validation of reference genes for gene expression studies in virus-infected *Nicotiana benthamiana* using quantitative real-time PCR. PLoS ONE.

[26] Rotenberg D, Thompson TS, German TL, Willis DK (2006). Methods for effective real-time RT-PCR analysis of virus-induced gene silencing. J Virol Methods.

[27] Wang Z, Gerstein M, Snyder M (2009). RNA-Seq: a revolutionary tool for transcriptomics. Nature Rev.

[28] Ansorge WJ (2009). Next genenration DNA sequencing techniques. New Biotechnol.

[29] Haas BJ, Zody MC (2010). Advancing RNA-Seq analysis. Nature Biotechnol.

[30] Wang L, Li P, Brutnell TP (2010). Exploring plant transcriptome using ultra high-throughput sequencing. Brief Funt Genomics.

[31] Pombo MA, Zheng Y, Fei Z, Martin GB, Rosli HG (2017). Use of RNA-seq data to identify and validate RT-qPCR reference genes for studying the tomato-*Pseudomonas* pathosystem. Sci Rep.

[32] Huang HC (1988). Molecular cloning of a *Pseudomonas syringae* pv. *syringae* gene cluster that enables *Pseudomonas fluorescens* to elicit the hypersensitive response in tobacco plants. J Bacteriol.

[33] Cuppels DA (1986). Generation and characterization of Tn5 insertion mutations in *Pseudomonas syringae* pv. *tomato*. Appl Environ Microbiol.

[34] Wei CF (2007). A *Pseudomonas syringae* pv. *tomato* DC3000 mutant lacking the type III effector HopQ1-1 is able to cause disease in the model plant *Nicotiana benthamiana*. Plant J.

[35] Kozera B, Rapacz M (2013). Reference genes in real-time PCR. J Appl Genet.

[36] Vandesompele J (2002). Accurate normalization of real-time quantitative RT-PCR data by geometric averaging of multiple internal control genes. Genome Biol.

[37] Hellemans J, Mortier G, De Paepe A, Speleman F, Vandesompele J (2007). qBase relative quantification framework and software for management and automated analysis of real-time quantitative PCR data. Genome Biol.

[38] Andersen CL, Jensen JL, Ørntoft TF (2004). Normalization of real-time quantitative reverse transcription-PCR data: a model-based variance estimation approach to identify genes suited for normalization, applied to bladder and colon cancer data sets. Cancer Res.

[39] Pfaffl MW, Tichopad A, Prgomet C, Neuvians TP (2004). Determination of stable housekeeping genes, differentially regulated target genes and sample integrity: BestKeeper – Excel-based tool using pair-wise correlations. Biotechnol Lett.

[40] Xie F, Xiao P, Chen D, Xu L, Zhang B (2012). miRDeepFinder: a miRNA analysis tool for deep sequencing of plant small RNAs. Plant Mol Biol.

[41] Lacerda ALM (2015). Reference gene selection for qPCR analysis in tomato-bipartite Begomovirus interaction and validation in additional tomato-virus pathosystems. PLoS ONE.

[42] Wang Q (2012). Stability of endogenous reference genes in postmortem human brains for normalization of quantitative real-time PCR data: comprehensive evaluation using geNorm, NormFinder, and BestKeeper. Int J Legal Med.

[43] Robledo D (2014). Analysis of qPCR reference gene stability determination methods and a practical approach for efficiency calculation on a turbot (*Scophthalmus maximus*) gonad dataset. BMC Genom.

[44] Zhang W-X (2016). Selection of suitable reference genes for quantitative real-time PCR normalization in three types of rat adipose tissue. Int J Mol Sci.

[45] Scholtz JJ, Visser B (2013). Reference gene selection for qPCR gene expression analysis of rust-infected wheat. Physiol Mol Plant Pathol.

[46] Miranda VdJ (2013). Validation of reference genes aiming accurate normalization of qPCR data in soybean upon nematode parasitism and insect attack. BMC Res Notes.

[47] Fang P (2015). Assessment of reference gene stability in Rice stripe virus and Rice black streaked dwarf virus infection rice by quantitative Real-time PCR. Virol J.

[48] Rosli HG, Martin GB (2015). Functional genomics of tomato for the study of plant immunity. Brief Funct Genomics.

[49] González-Agüero M (2013). Identification of two putative reference genes from grapevine suitable for gene expression analysis in berry and related tissues derived from RNA-Seq data. BMC Genom.

[50] Yim AK-Y (2015). Using RNA-seq data to evaluate reference genes suitable for gene expression studies in soybean. PLoS ONE.

[51] Mariot RF (2015). Selection of reference genes for transcriptional analysis of edible tubers of potato (*Solanum tuberosum* L.). PLOS ONE.

[52] Ma R, Xu S, Zhao Y, Xia B, Wang R (2016). Selection and validation of appropriate reference genes for quantitative real-time PCR analysis of gene expression in *Lycoris aurea*. Front Plant Sci.

[53] Muller OA (2015). Genome-wide identification and validation of reference genes in infected tomato leaves for quantitative RT-PCR analyses. PLoS ONE.

[54] Fernandez-Pozo N (2014). The Sol Genomics Network (SGN)—from genotype to phenotype to breeding. Nucleic Acids Res.

[55] Nguyen HP (2010). Methods to study PAMP-triggered immunity using tomato and Nicotiana benthamiana. Mol Plant-Microbe Interact.

