# Supplementary material for: Transcriptome-based identification and validation of reference genes for plant-bacteria interaction studies using Nicotiana benthamiana
Source: Sci Rep. 2019 Feb 7;9:1632. doi: 10.1038/s41598-018-38247-2 (PMC6367355; doi:10.1038/s41598-018-38247-2)
Supplement: Supplementary file 1 — Supplementary information [file 41598_2018_38247_MOESM1_ESM.pdf]

Manuscript tittle

Transcriptome-based identification and validation of reference genes for plant-bacteria interaction studies using *Nicotiana benthamiana*

List of authors:

1. Marina A. Pombo
2. Romina N. Ramos
3. Yi Zheng
4. Zhangjun Fei
5. Gregory B. Martin
6. Hernan G. Rosli

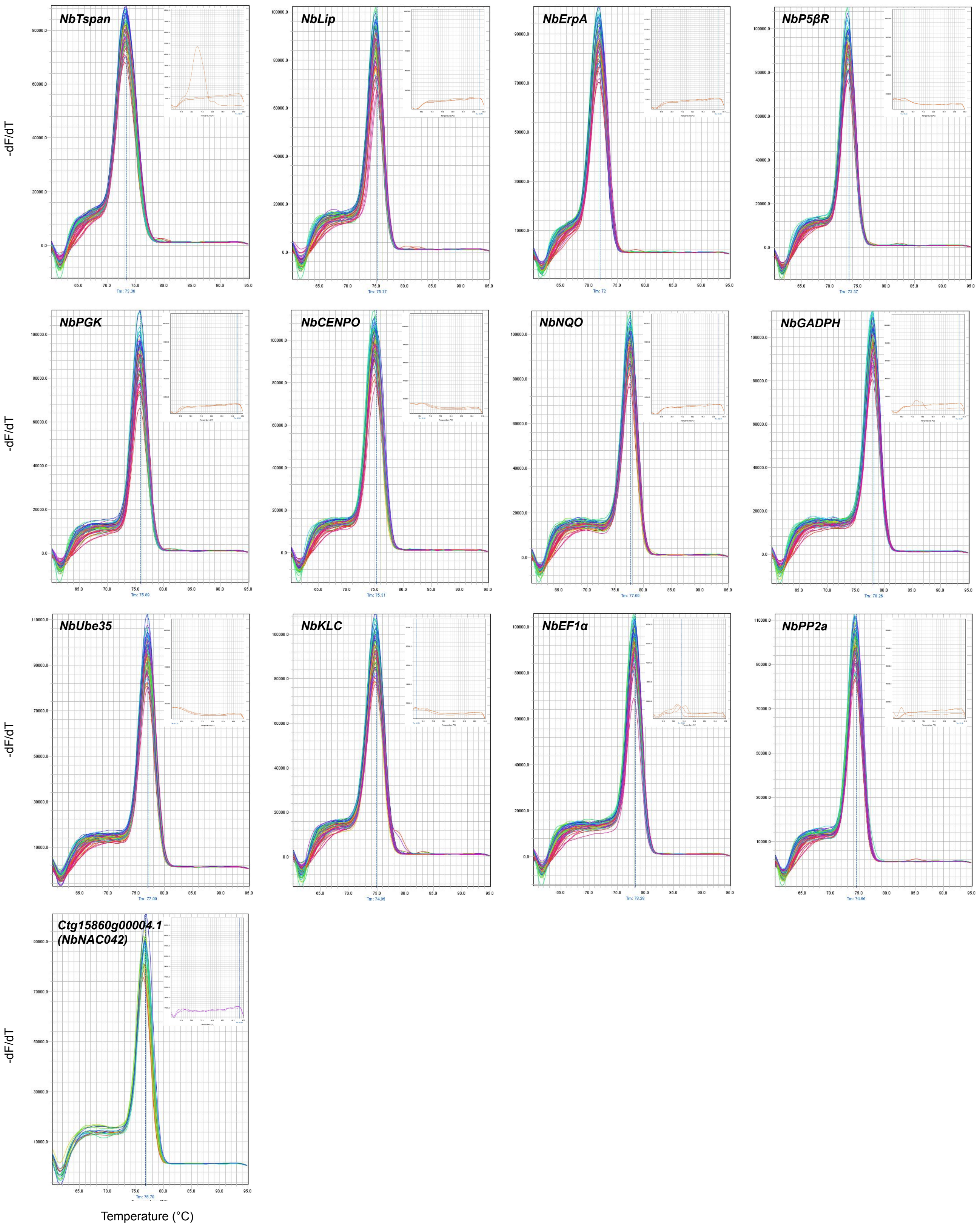

Supplementary Fig. S1: Validation of primer pairs of *N. benthamiana* candidate reference genes for RT-qPCR experiments and *NbNAC042*. PCR amplification specificity was measured by the presence of unique amplicons using melting curve analysis. Negative sample (water templates) melting curves performed for each analyzed gene are shown in a small graph in the upper right corner.

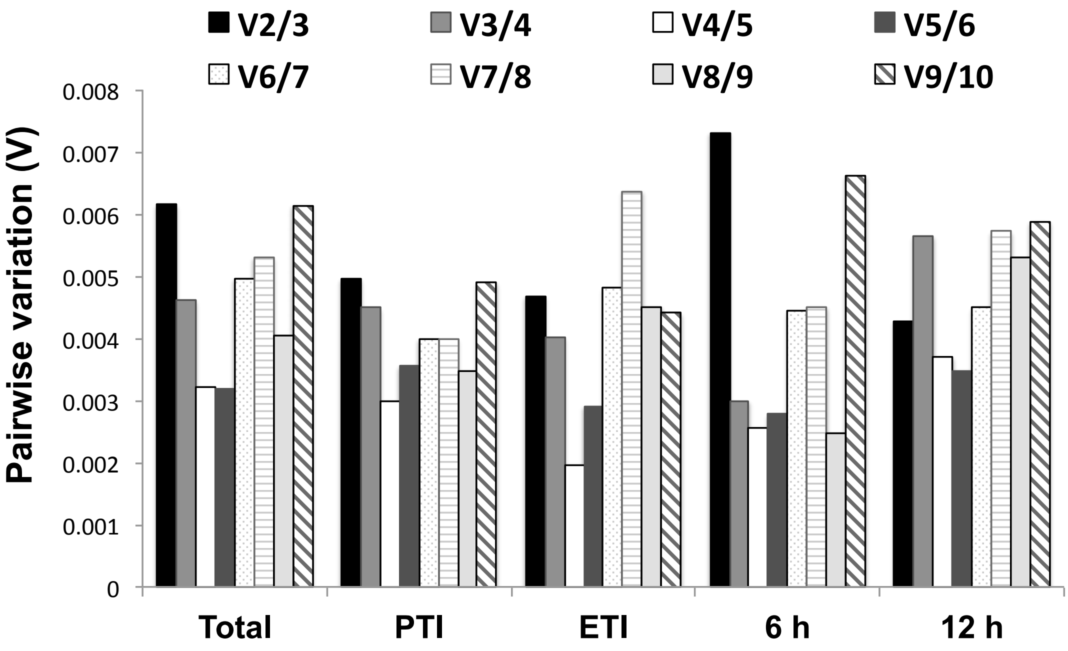

Supplementary Fig. S2: Pairwise variation (Vn/Vn+1) analysis for the determination of the optimal number of reference genes. *N. benthamiana* reference genes were included in the analysis using the final ranking order shown in Table 3. The pairwise variation was calculated considering all the samples together (Total), mock and *P. fluorescens* inoculations (PTI), *Pst* DC3000 and *Pst* DC3000 Δ*hopQ1-1* (ETI), samples taken at 6 hpi (6 h) or samples taken at 12 hpi (12 h).
